# An illustrated key to the species of the genus *Narella* (Cnidaria, Octocorallia, Primnoidae)

**DOI:** 10.3897/zookeys.822.29922

**Published:** 2019-02-04

**Authors:** Stephen D. Cairns, Michelle L. Taylor

**Affiliations:** 1 Department of Invertebrate Zoology, National Museum of Natural History. Smithsonian Institution, Washington, DC, 20560, USA Smithsonian Institution Washington United States of America; 2 School of Biological Sciences, University of Essex, Wivenhoe Park, Colchester, CO4 3SQ, UK University of Essex Colchester United Kingdom

**Keywords:** Alcyonacea, Calcaxonia, dichotomous key, Primnoidae, tabular key

## Abstract

A history of the description of the 50 valid species of *Narella* is given, beginning with the first species described in 1860. To help differentiate the various species, a tabular and a polychotomous key are provided. The species in the keys are arranged using nine characters or character sets that are believed to be of value at the species level. New characters or new significance given to previously described characters used in our keys include: 1) the nature of the dorsolateral edge of the basal scale, being ridged or not, 2) the thickness of the body wall scales, and 3) the arrangement of the coenenchymal scales (imbricate or mosaic), their thickness (thin or massive), and their outer surface ornamentation (ridged or not). All characters used in the keys are illustrated.

## Introduction

The first species of *Narella* was described as *Primnoaregularis* by Duchassaing and Michelotti (1860) collected off Guadeloupe, Lesser Antilles at an unknown depth. This is somewhat remarkable in that 366 m is the shallowest depth from which this species is known, and it was thus collected at a time when deep-water animals were not thought to occur below approximately 200 m. *Primnoaregularis* was made the type (by monotypy) of the newly described genus *Narella* by Gray (1870), calling it that name perhaps because the polyps resembled a series of small noses (Latin *naris* = nostril). The holotype is deposited at the Turin Museum (Volpi and Benvenuti 2003) but because of its poor condition was set aside to be replaced by a neotype (Cairns and Bayer 2004; ICZN 2005).

The next species to be described in the genus, *Stachyodesregularis* Wright & Studer, 1889, from the Kermadec Islands, was unfortunately also called *regularis*, but placed in the newly described genus *Stachyodes* Wright & Studer, 1887 in Studer (1887), a junior synonym of *Narella*. Because Versluys (1906) considered it and *P.regularis* of Duchassaing and Michelotti (1860) to be in the same genus, the Wright & Studer species was thought to be a junior homonym and thus it required a new name, which he gave as *S.studeri* Versluys, 1906. It also became the type species of *Stachyodes*. Yet another genus name that was subsequently synonymized with *Narella* was proposed by Wright and Studer (1889) as *Calypterinus*, the type species being *C.allmani* Wright & Studer, 1889 (Fiji).

In the first of several species to be described based on specimens collected by the US Fish and Wildlife Service vessel *Albatross*, Studer (1894) described *Stachyodes* (= *Narella*) *ambigua* from off the Galapagos Islands.

Next followed Versluys’ (1906) beautifully illustrated and finely described revision of the deep-water octocorals of the *Siboga* Expedition from Indonesia, which included the description of seven new species, all of which he also placed in *Stachyodes*. This work set the standard for future morphological descriptions within the genus.

In the next ten years a flurry of new species were described from around the world: four from off Japan (Kinoshita 1907), one from off Sumatra (Kükenthal 1907), one from the Hawaiian Islands (Nutting 1908), three from the North Atlantic (Hickson 1909; Kükenthal 1912, 1915), and one from the southwest Indian Ocean (Thomson 1911). *Narellaelegans* Tixier-Durivault & Lafargue, 1968 is believed to be a junior synonym of *N.versluysi* (Hickson, 1909), originally described in Stephens and Hickson (1909). Thomson also described a species from the southwest Indian Ocean, *S.capensis* Thomson, 1917, which was later synonymized with *N.gilchristi* (Thomson, 1911). But most notable from this time period was Kükenthal’s (1919) report on the deep-water octocorals of the *Siboga* expedition, in which he re-described all the species of *Narella* (as *Stachyodes*) and provided a morphological key to the 18 valid species. One hundred years later these are still the characters used to discriminate species and form the basis for the keys presented herein.

Aurivillius (1931) described one new species from off Japan, and Deichmann (1936) two new species from the northwest Atlantic Ocean. Finally, the “modern” era of *Narella* taxonomy was introduced by Bayer (1951), who finally synonymized *Stachyodes* and *Calypterinus* with *Narella*, and also described a new species from Indonesia. He later described two new species from off the Hawaiian Islands (Bayer 1995, 1997), one of them, *N.nuttingi* Bayer, 1997, later being synonymized with *N.dichotoma* (Cairns & Bayer, 2007). In collaboration with Cairns, Bayer also revised the *Narella* species from the northwest Atlantic (Cairns and Bayer 2003), describing two new species, and from the Hawaiian Islands (Cairns and Bayer 2007), describing six new species. They subsequently also placed the genus in phylogenetic perspective in a morphology-based cladogram, and listed the 38 known species at that time (Cairns and Bayer 2009). Also in 2007, Cairns and Baco (2007) described five new species from deep seamounts in the Gulf of Alaska.

Cairns described five more new species from the New Zealand region (Cairns 2012) and six from the northern and central Pacific (Cairns 2018), which prompted the need for this synthetic key to the species. Cairns (2018) also made one previously described species of *Narella*, *N.mesolepis* Cairns, 2012, the basis for a new genus, *Pseudonarella*. Taylor and Rogers (2015) placed *Narella* in a phylogenetic perspective using molecular data, and listed the 44 species known at that time, although *S.regularis* should be considered as junior synonym of *N.studeri*, and Cairns (2018) considered *N.irregularis* to be a junior synonym of *N.horrida*. Finally, Taylor and Rogers (2017) described three new species from the southwest Indian Ocean, and listed all species known at that time.

The genus *Narella* represents a highly successful adaptive radiation within the primnoids and more species are expected to be discovered. This is the reason why we here present two keys (a tabular and polychotomous key), the first since Kükenthal’s (1919) work, i.e., to facilitate comparison of species for identification purposes, and to examine this genus before new species are described.

## Materials and methods

Many of the descriptions and diagnoses are based on original literature, which is duly cited. Descriptive terms used are found in the trilingual glossary of Bayer et al. (1983). Reviewing holotypes involved preparing sclerites for viewing under a light microscope following procedures well-documented elsewhere (Alderslade 1998; Fabricius and Alderslade 2001; Cairns 2016).

## Taxonomy

### Subclass Octocorallia

#### Order Alcyonacea

##### Suborder Calcaxonia

###### Family Primnoidae Milne Edwards, 1857

####### Genus Narella

Taxon classificationAnimaliaAlcyonaceaPrimnoidae

Gray, 1870

Narella Gray, 1870: 49; Cairns and Bayer 2009: 43.
Stachyodes Wright & Studer in Studer 1887: 49.
Calypterinus Wright & Studer in Studer 1887: 49–50.

######## Diagnosis.

Colonies branched dichotomously (laterally or equal), pinnately, in a lyrate fashion, or unbranched. Polyps arranged in whorls, all polyps facing downward in contracted condition. Each polyp covered with three (rarely four) pairs of abaxial body wall scales (i.e., one pair of basals, one or rarely two pairs of medials, and one pair of buccals) and a variable number of pairs of smaller adaxial scales, nonetheless leaving the adaxial face largely naked. Articular ridge not present on basal scales. Paired infrabasal scales often present. Opercular scales keeled on inner surface. Coenenchymal scales thin and imbricate or thick and mosaic in placement, and sometimes prominently ridged.

######## Type species.

*Primnoaregularis* Duchassaing & Michelotti, 1860, by monotypy.

######## Discussion.

Currently there are 50 valid species in the genus *Narella*, the most speciose in the family Primnoidae (Taylor and Rogers 2015). The species in both keys (tabular (Table 1) and polychotomous, below) are roughly presented in an order that follows the major characters as outlined below, these characters we purport to be valuable in the distinction of species of *Narella*.

**Table 1.** Tabular Key to the species of the genus *Narella*.

Species	Dorsolateral edge of basal scale	Pairs of body wall scales	Polychaete commensalism	Branching mode	Body wall scale thickness	Coenenchymal scales: imbricate, thickness; ridged	Polyps/whorl; whorl diameter (mm)	Polyp length (mm)	Distal edge of basal scales	Other characters	Geographic and depth range
*N.macrocalyx* Cairns & Bayer, 2007	Small ridge	3	Present	Sparse,	Thin	Thin, imbricate; rarely ridged	4–6; 7–11	4.5–5.5	Lobate, smooth		Hawaiian Islands, 1206–1807 m
*N.gilchristi* (Thomson, 1911)	Small ridge	3	Present	lyrate, secondarily dichotomous	Thin	Thick, mosaic; unridged	4–8; 4–9	2––3	Lobate, smooth		Southwest Indian Ocean, 90–1365 m
*N.ferula* Cairns, 2018	Multi-ridged	3	Absent	Unbranched	Thin	Thin, imbricate; ridged	2–3; 3.6	2.3–2.5	Serrate cowl, spurs	Medial scales also with serrate margin	Palmyra Atoll, 1023 m
*N.hawaiinensis* Cairns & Bayer, 2007	Inconspicuous basal ridge	3	Absent	Unbranched	Thin	Thin, imbricate; ridged	3–5; 5–6	3.4–4.1	Lobate, smooth		HI, Johnston Atoll, 1492–1944 m
*N.muzikae* Cairns & Bayer, 2007	Multi-ridged	3	Absent	Common coenosteum (bolus)	Thin	Thin, imbricate; ridged	3–6; 3–4	1.7–2.2	Lobate, serrate	Base strongly calcified	Hawaiian Islands, 326–381 m
*N.merga* Cairns, 2018	Two ridges basally	3	Absent	Y-shaped	Thin	Thin, imbricate; ridged	3; 4.4	4	Lobate (short cowl)		Wake Island, 2575 m
*N.fordi* Cairns, 2018	Multi-ridged	3	Absent	Sparse, equal dichotomous	Thin	Thin, imbricate; ridged	3;3.4–3.5	2.1–2.6	Lobate, smooth	Medial scales ridged	Phoenix Islands, 1899 m
*N.cristata* Cairns & Baco, 2007	Single ridge	3	Absent	Sparse, equal dichotomous	Thin	Thin, imbricate; ridged (sail scales)	2–4; 3.4	2.1–3.0	Lobate, smooth	Medial and buccals ridged; occasionally four pairs of bw scales	Gulf of Alaska seamounts, 3385 m
*N.alvinae* Cairns & Bayer, 2003	Single ridge	3	Absent	Sparse, equal dichotomous	Thin	Thin, imbricate; ridged	4; 3.8	2.7–3.1	Lobate. smooth	Medial scales elongate	Bermuda, 3419 m
*N.bayeri* Cairns & Baco, 2007	Single ridge	3	Absent	Sparse, equal dichotomous	Thin	Thin, imbricate; ridged (sail scales)	5–7; 3.5	2.2–3.4	Lobate, smooth	Medial scales ridged	Gulf of Alaska seamounts, 3277–4091 m
*N.alaskensis* Cairns & Baco, 2007	Low ridge	3	Absent	Sparse, equal dichotomous	Thin	Thin, imbricate; ridged (sail scales)	5–9; 7.5	2.7–3.2	Lobate (narrow), smooth	Medial scales ridged	Gulf of Alaska seamounts, 2377–3075 m
*N.arbuscula* Cairns & Baco, 2007	Tall, short ridge	3	Absent	Sparse, equal dichotomous	Thin	Thin, imbricate; ridged (sail scales)	6–7; 6.8	3.4–4.7	Lobate, smooth	Whorls crowded	Gulf of Alaska seamounts, 2775–3465 m
*N.pauciflora* Deichmann, 1936	Multi-ridged	3	Absent	Equal dichotomous	Thin	Thin, imbricate; complex ridging	2–5; 4	2.6–2.8	Lobate, smooth	Adaxial buccals as ridged ascus scales	Northwest Atlantic, 738–1473 m
*N.bowersi* (Nutting, 1908)	One ridge basally	3	Absent	Equal dichotomous	Thin	Thin, imbricate; ridged	3–4; 4.5	2.5–3.2	Tall, serrate	Buccal scales serrate	Hawaiian islands, 1218–1758 m
*N.gaussi* (Kükenthal, 1912)	Multi-ridged	3	Absent	Equal dichotomous	Thin	Thin, imbricate; ridged	4–5; 3	2.1–3.0	Lobate (low), smooth	Radial ridges on all body wall scales	Antarctica, 2450 m
*N.parva* (Versluys, 1906)	Multi-ridged	3	Absent	Equal dichotomous	Thin	Thin, imbricate; ridged	4–6; 2.5–3.2	2.0–2.4	Tall, narrow, smooth	Adaxial buccal scales ridged	Southwest Pacific, 920–2400 m
*N.regularis* (Duchassaing & Michelotti., 1860)	Multi-ridged	3	Absent	Equal dichotomous	Thin	Thin, imbricate; ridged	4–5; 3.2	2.0–2.3	Lobate, smooth	Medial and buccals ridged	Northwest Atlantic, 366 – 792 m
*N.valentine* Taylor & Rogers, 2017	One tall ridge	3	Absent	Lyrate, secondarily dichotomous	Thin	Thin, imbricate; flat	4–5; 2.4–2.8	1.5–1.8	Tooth-like apex	Medials ridged	Southwest Indian Ocean, 383–444 m
*N.virgosa* Cairns, 2018	Multi-ridged	3	Absent	Lyrate, secondarily dichotomous and bushy	Thin	Thin, imbricate; ridged (sail scales)	3–4; 3.3–4.2	2.6–2.8	Lobate, smooth	Medials and buccals ridged	Hawaiian Islands and Johnston Atoll; 1901–1985 m
*N.bellissima* (Kükenthal, 1915)	Low ridge basally	3	Absent	Lyrate, secondarily dichotomous	Thin	Thin, imbricate; ridged (sail scales)	3–8; 3.15	2.0–2.2	Lobate, smooth		Amphi-Atlantic, 161–1968 m
*N.ornata* Bayer, 1995	Multi-ridged	3	Absent	Unknown	Thin	Thin, imbricate; ridged	3–4; 3.5	3	Serrate distal margin	All scales, including adaxial buccals, radially ridged	Hawaiian Islands, 748–1007 m
*N.spectablis* Cairns & Bayer, 2003	One tall ridge	4	Absent	Unbranched	Thin	Thin, imbricate; ridged (sail scales)	3; 2.8	3.5	Lobate, smooth (low)	All body wall scales ridged	Bahamas, 1485 m
*N.abyssalis* Cairns & Baco, 2007	Multi-ridged	4	Absent	Sparse, dichotomous	Thin	Thin, imbricate; ridged (sail scales)	2–4; 2.8	1.9–2.4	Lobate, smooth (low)	All body wall scales ridged	Gulf of Alaska seamounts, 4594 m
*N.laxa* Deichmann, 1936	Absent	4	Absent	Equal dichotomous	Thin	Thin, imbricate; multiple ridges	3–5; 3.6	3	Lobate, smooth	3 pairs of adaxial buccal scales	Amphi-North Atlantic, 2980–3186 m
*N.horrida* (Versluys, 1906)	Absent	3	Present	From common bolus	Massive	Thick, mosaic; unridged	5–6; 6–9	2.0–3.4	Spinose (massive)	Medial scales also spinose	Indonesia, 204 m
*N.hypsocalyx* Cairns, 2012	Absent	3	Present	From common bolus	Thin	Thin, imbricate; unridged	9; 13	2.7	Tall and serrate	Adaxial buccals elongate	New Zealand, 510–1118 m
*N.clavata* (Versluys, 1906)	Absent	3	Present	Sparse, dichotomous	Massive	Thick, mosaic; unridged	4–14; 7–8	2––3	Tall, narrow, smooth	Adaxial buccals numerous	Indonesia, Philippines, 128–335 m
*N.ambigua* (Studer, 1894)	Absent	3	Present	Sparse, dichotomous	Thin	Thick, mosaic; unridged	5–7; 6–7	2.5–3.0	Lobate, tall, smooth	3 pairs adaxial buccals	Galapagos, Gulf of Panama, 702– 1463 m
*N.aurantiaca* Cairns, 2018	Absent	3	Present	Sparse, dichotomous	Thin	Thin, interlocking; ridged	4–6; 6.5–7.0	2.8–3.2	Lobate, smooth		Wake Island, 745 m
*N.leilae* Bayer, 1951	Absent	3	Present	Sparse, dichotomous	Thin	Thin, imbricate; ridged (sail scales)	4–6; 5.2–5.6	2.0–2.5	Serrate cowl	Edges of buccals undulate	Indonesia, 740 m
*N.alata* Cairns & Bayer, 2007	Absent	3	Present	Equal dichotomous	Thin	Thin, imbricate; medial scale	4–5; 4–5	2.5–3.1	Lobate, tall (cowl), smooth	Whorls closely spaced	Hawaiian Islands, 477–750 m
*N.vermifera* Cairns & Bayer, 2007	Absent	3	Present	Equal dichotomous	Thin	Thick, mosaic; very low ridges	3–5; 4	1.8–2.0	Lobate, tall, smooth	Buccals in closed position	Hawaiian Islands, 275–527 m
*N.allmani* (Wright & Studer, 1889)	Absent	3	Present	Equal dichotomous	Thin	Thick, mosaic; unridged	4–7;5	3	Tall, serrate		Fiji, depth unknown
*N.obscura* (Versluys, 1906)	Absent	3	Present	Equal dichotomous	Thin	Thick, mosaic; unridged	4–6; 6–7	2.7–2.8	Lobate (undulate), smooth (cowl)		Indonesia, 984 m
*N.dampieri* Cairns, 2012	Absent	3	Present	Equal dichotomous	Thin	Thick, mosaic; unridged	5–8; 7	1.4–1.9	Lobate, tall, narrow	Numerous adaxial buccal scales	Lord Howe Islands, 342 m
*N.mosaica* Cairns, 2012	Absent	3	Present	Equal dichotomous	Massive	Thick, mosaic; unridged	3–5; 5–6	2.7–3.1	Lobate, slender, smooth		New Zealand, 228–294 m
*N.vulgaris* Cairns, 2012	Absent	3	Present	Equal dichotomous	Massive	Thick, mosaic; unridged	4–6; 4–5	2.0–2.4	Lobate, smooth	2 pairs adaxial buccals are ridged	New Zealand, 335–1165 m
*N.orientalis* (Versluys, 1906)	Absent	3	Present	Unknown	Thin	Thin, imbricate; unridged (concave)	6; 5.8	2.2–3.0	Lobate, smooth		Indonesia, 520 m
*N.calamus* Cairns, 2018	Absent	3	Absent	Unbranched	Thin	Thin, imbricate; ridged (sail sacles)	4; 5	4.5–5.0	Serrate, blunt		Wake Island, 2073 m
*N.versluysi* (Hickson, 1909)	Absent	3	Absent	Unbranched or very sparsely	Thin	Thin, imbricate; medial ridge	4–7; 5–7	3.2–3.7	Lobate, smooth	Basal scale ridged internally	Amphi-North Atlantic, 550–3100 m
*N.speighti* Taylor & Rogers, 2017	Absent	3	Absent	Sparse, dichotomous	Thin	Thin, imbricate; unridged	3–4; 2.5–3.6	2.0–2.2	Lobate (slender), smooth		Southwest Indian Ocean, 870 m
*N.grandiflora* (Kükenthal, 1907)	Absent	3	Absent	Sparse, dichotomous	Thin	Thick, mosaic; unridged	4–5; 4.5	3	Lobate, smooth	Numerous adaxial buccal scales	Indonesia, 805 m
*N.studeri* (Versluys, 1906)	Absent	3	Absent	Equal dichotomous	Massive	Thick, mosaic; unridged	4–8; 4–5	3.0–3.3	Lobate, smooth	Smooth body wall scales	New Zealand, Indonesia, 732–1392 m
*N.biannulata* (Kinoshita, 1907)	Absent	3	Absent	Equal dichotomous	Massive	Thick, mosaic; unridged	6–7; 4.8	1.8–2.0	Lobate, smooth	Adaxial buccals absent; medial scales closed	Japan, depth unknown
*N.candidae* Taylor & Rogers, 2017	Absent	3	Absent	Equal dichotomous	Thin	Thick, mosaic; unridged (smooth)	4–6; 4–5	2.0–2.4	Lobate, smooth		Southwest Indian Ocean, 763 m
*N.japonensis* (Aurivillius, 1931)	Absent	3	Absent	Equal dichotomous	Thin	Thin, imbricate; unridged	3–6; 3.5–4.0	2––3	Lobate, smooth	Stem stiff	Japan, 732 m
*N.gigas* Cairns & Bayer, 2007	Absent	3	Absent	Equal dichotomous	Thin	Thin, imbricate; ridged	10–14; 9–12	2.5–3.0	Lobate, tall, narrow, smooth		Hawaiian Islands, 362–399 m
*N.dichotoma* (Versluys, 1906)	Absent	3	Absent	Equal dichotomous	Thin	Thin, imbricate; low ridges	3–5; 4–5	2.8–3.1	Lobate, smooth		Hawaiian Islands, Malaysia, 204–1448 m
*N.megalepis* (Kinoshita, 1908)	Absent	3	Absent	Equal dichotomous	Thin	Thin, imbricate; ridged	5–8; 6–7	2.5–3.0	Lobate, smooth	Numerous small adaxial buccal scales	Japan, depth unknown
*N.compressa* (Kinoshita, 1908)	Absent	3	Absent	Lyrate	Massive	Thick, mosaic; unridged	7–8; 3	2	Lobate, smooth		Japan, Phoenix Islands, 501 m

**Figure 1.**
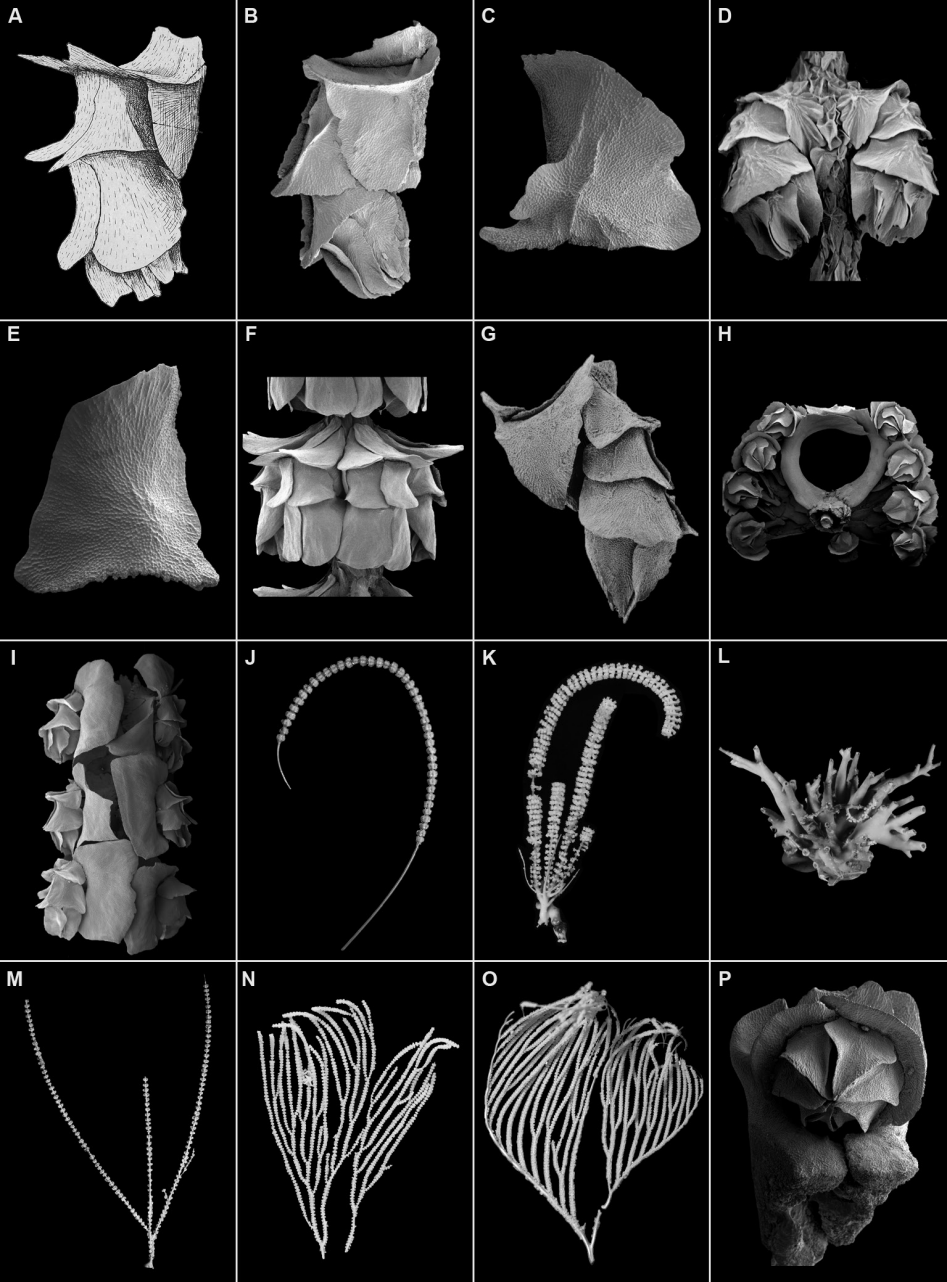
**A, B** lateral view of a polyp showing dorsolateral ridge for entire height of basal scale (**A***N.parva* from Versluys (1906)**B***N.bayeri*) **C** basal scale of *N.hawaiinensis* showing dorsolateral ridge only on lower half of scale **D** whorl of polyps of *N.pauciflora* showing multiple dorsolateral ridges on the basal scales **E** basal scale of *N.vulgaris* showing the lack of a dorsolateral ridge, and a lobate distal edge **F** polyp whorl of *N.bellissima* showing the three pairs of body wall scales **G** polyp of *N.laxa* having four pairs of body wall scales **H** polyp whorl of *N.hypsocalyx* showing highly modified basal scales forming a cross section view of a cylindrical worm tube **I** polyp whorl of *N.vulgaris* showing highly modified basal scales forming a lateral view of a cylindrical worm tube **J** unbranched colony of *N.versluysi***K, L** branching from a basal bolus of *N.hypsocalyx***M** sparse, dichotomous branching of *N.macrocalyx***N** equal, dichotomous branching of *N.vulgaris***O** lyrate branching of *N.bellissima***P** massive basal scales of *N.clavata*.

***Dorsolateral edge of basal scale ridged or not ridged***: The dorsolateral edge (the point of inflexion of the scale from the dorsal region to the lateral region) of the basal scale is consistently ridged or not ridged (Fig. 1E) in each species, with the only exception of *N.macrocalyx*, which is inconspicuously ridged, and sometimes (rarely) lacks the ridge. This external ridging may help give strength to basal sclerites. The ridge may be single and extend from the base to the tip of the scale (Fig. 1A, B), or partial, occurring only at the base of the scale (Fig. 1C). Or, there may be multiple short ridges occurring in this region of the scale (Fig. 1D). The ridges may be tall or low. This character is relatively easy to observe, but usually requires the removal of a polyp from a whorl, drying the specimen, and then applying a dye to help see the characteristic ridging structure.

***Number of pairs of body wall scales***: Most species of *Narella* have three pairs of abaxial body wall scales (basal, medial, and buccal, Fig. 1F), but in three species there is consistently an extra pair of medial scales (Fig. 1G). Also, specimens of some species that have otherwise three pairs of body wall scales will have occasional polyps with four pairs of body wall scales. This is a fairly easily observed character when using a dissecting microscope.

***Worm commensalism***: The commensal association with a polychaete worm, usually a polynoid (Cairns and Bayer 2008, Cairns 2012, Britayev et al. 2014, Serpetti et al. 2017), is considered to be characteristic of the species, and is easily observed even without a microscope. The facing basal scales of two adjacent polyps are greatly enlarged and modified (reflexed) in order to make an elongate cylindrical tube for the worm (Fig. 1H, I).

***Branching mode***: The mode of branching, and thus colony shape, is considered to be characteristic of the species. Modes include: unbranched (Fig. 1J), branching from a common basal coenenchyme or bolus (Fig. 1K, L), sparse equal dichotomous branching (Fig. 1M), equal dichotomous branching (Fig. 1N), and lyrate (Fig. 1O), which is often followed by dichotomous branching. Lyrate branching might be considered as a special case of dichotomous branching in which the outer component of each bifurcation maintains a straight line while the inner branches remain roughly parallel to one another.

**Figure 2.**
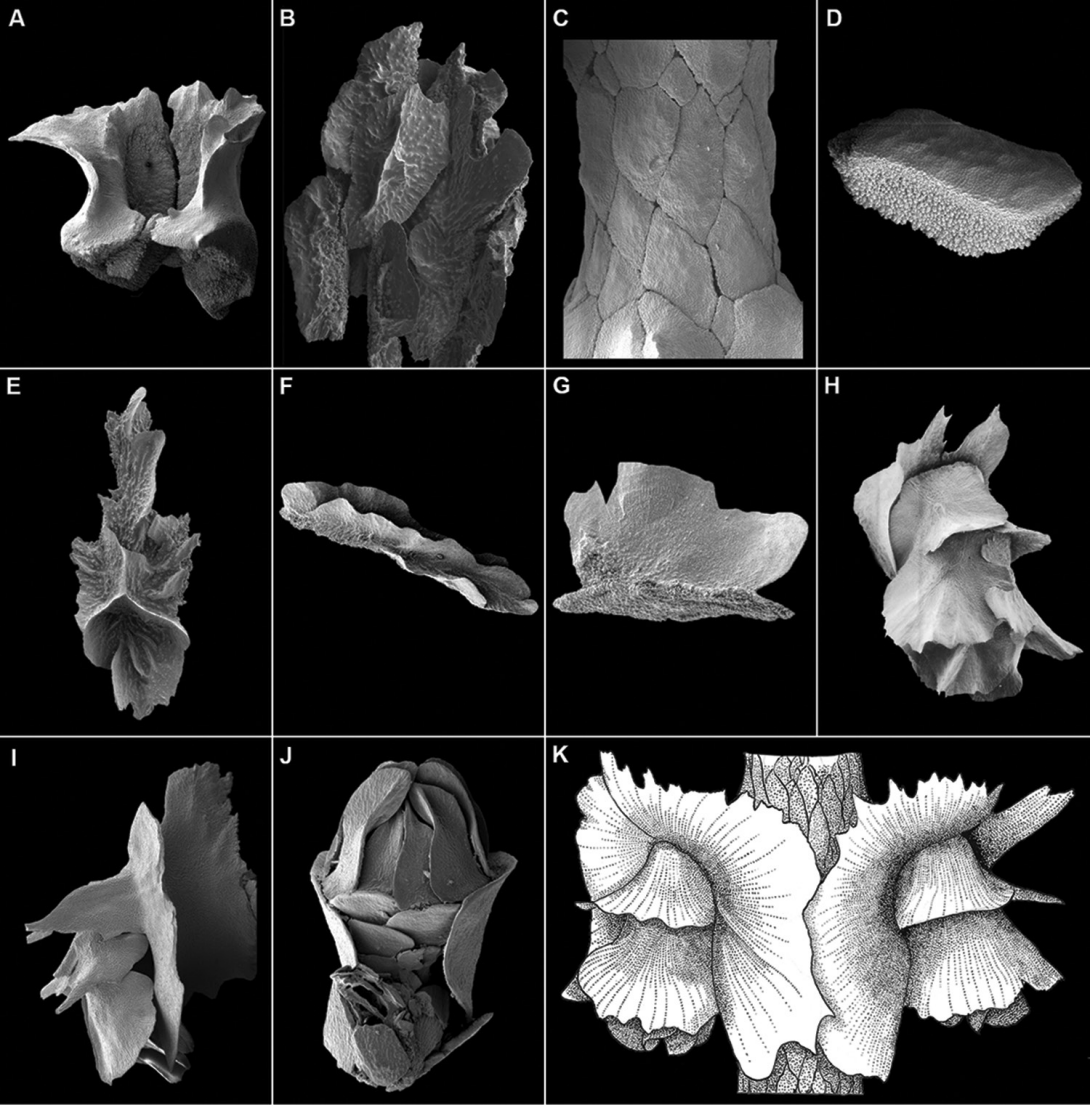
**A** massive basal scales of *N.clavata***B** thin, imbricate coenenchymal scales of *N.fordi***C** thick, mosaic arranged coenenchymal scales of *N.mosaica***D** individual thick coenenchymal scale of *N.mosaica* with a finely granular outer surface **E** complexly ridged coenenchymal scale of *N.muzikae***F** single medial coenenchymal ridge of *N.pauciflora***G** sail scale of *N.spectabilis***H** serrate distal margin of body wall scales of *N.bowersi***I** spinose body wall scales of *N.horrida***J** adaxial body wall scales of *N.dampieri***K** polyp pair of *N.leilae* showing extensive cowl and serrate distal edges of body wall scales (from Bayer, 1951).

***Body wall scale thickness***: In some species the body wall scales are quite thick, or massive (Figs 1P, 2A). This trait is often correlated with having thick coenenchymals as well (see next character). This character is best seen using scanning electron microscopy of individual sclerites.

***Coenenchymal scales arrangement and ornamentation***: The coenenchymal scales of most species are relatively thin, having the same thickness as a body wall scale, and have edges that slightly overlap those of other adjacent coenenchymal scales (Fig. 2B). But some species have quite thick scales (Fig. 2C–D) that are so massive that they cannot overlap adjacent scales and thus produce a mosaic, polygonal, or tessellate pattern, also called “cobblestone” (Williams 1992). The term mosaic is used herein. Coenenchymal scales usually have a finely granular outer surface (Fig. 2D), but many species have scales that bear a single longitudinal (Fig. 2F) or multiple complexly arranged ridges (Fig. 2E). If these ridges are quite tall they have been termed sail scales (Cairns 2016)(Fig. 2G). Mosaic coenenchymals are not usually ridged (Fig. 2D). This character is best seen using SEM.

***Polyps/whorl*; *whorl diameter***: Although every specimen and species has a range of polyps/whorl and whorl diameter, sometimes these numbers help to differentiate species. This character is easily determined using a dissecting microscope.

***Polyp length***: As above, this character has a range for every specimen and species, but can sometimes differentiate among species. The polyp length is essentially the horizontal length of the polyp, which consist of the length of the buccal scale and whatever part of the operculars protrude from the buccal scale. This character is easily determined using a dissecting microscope.

***Shape of the distal edge of basal scales***: The distal edge of the basal scales are usually slightly lobate and smooth (Fig. 1E), but in some species are serrate (Fig. 2H, K) or even spinose (e.g., *N.horrida*, Fig. 2I). It may extend far beyond its junction with the proximal edge of the medial scales as a cowl (Fig. 2K) or be quite short (Fig. 1D). This character is also easily determined using a dissecting microscope.

***Other characters***: Other characters that are used to describe and differentiate species but are not consistently addressed in the keys include: shape and number of adaxial body wall scales (Fig. 2J), external ridging of the medial and buccal scales, closure of the body wall rings, aspects of the opercular scales, body wall formula (i.e., ratio of length of basal: medial: buccal scales), and number of polyps per cm.

######## Geographic and depth range.

All ocean basins, 128–4594 m (Cairns 2012).

###### Polychotomous key to the species of the genus *Narella*

**Table:** 

1a	Dorsolateral edge of basal scale bears a longitudinal ridge or ridges (Fig. 1A, D)	**2**
1b	Dorsolateral edge of basal scale unridged (smooth) (Fig. 1F)	**9**
2a	Three pairs of body wall scales per polyp (Fig. 1A, B, F)	**3**
2b	Four pairs of body wall scales per polyp (Fig. 1G)	**18**
3a	Polychaete commensalism present, causing extreme modification of basal scales to form a tube (Fig. 1H, I)	**4**
3b	Polychaete commensalism absent (no tubes)	**5**
4a	Colony branching sparse (Fig. 1M); coenenchymal scales thin and imbricate in arrangement (Fig. 2B); Hawaiian Islands	*** N. macrocalyx ***
4b	Colony branching lyrate (Fig. 1O); coenenchymal scales thick and mosaic in arrangement (Fig. 2C, D); South West Indian Ocean	*** N. gilchristi ***
5a	Colonies unbranched (Fig. 1J)	**6**
5b	Branches of colony originate from a common base or from a basal bolus (Fig. 1K, L)	*** N. muzikae ***
5c	Branching in a Y-shape	*** N. merga ***
5d	Branching sparse, dichotomous (Fig. 1M)	**7**
5e	Branching equal, dichotomous (Fig. 1N)	**12**
5f	Branching lyrate, sometimes with subsequent dichotomous branching (Fig. 1O)	**16**
5g	Branching pattern unknown; all scales radially ridged	*** N. ornata ***
6a	Multiple ridges on dorsolateral edge of basal scales (Fig. 1D); polyps less than 2.5 mm in length	*** N. ferula ***
6b	Single inconspicuous ridge on dorsolateral edge of basal scales (Fig. 1A, B); polyps greater than 3.5 mm in length	*** N. hawaiinensis ***
7a	Polyps less than 4 mm in length	**8**
7b	Polyps more than 5 mm in length	**10**
8a	Multiple ridges on dorsolateral edge of basal scales	*** N. fordi ***
8b	Single ridge on dorsolateral edge of basal scales	**9**
9a	Buccal scales ridged (Fig. 1D); medial scales short; Gulf of Alaska	*** N. cristata ***
9b	Buccal scales unridged; medial scales elongate; Bermuda	*** N. alvinae ***
10a	Whorl diameter less than 4 mm	*** N. bayeri ***
10b	Whorl diameter greater than 6 mm	**11**
11a	Polyp length 3.4–4.7 mm	*** N. arbuscula ***
11b	Polyp length 2.7–3.2 mm	*** N. alaskensis ***
12a	Extremely few polyps per whorl (occasionally only two)	*** N. pauciflora ***
12b	More numerous polyps per whorl (up to six)(Fig. 1F)	**13**
13a	Whorl diameter greater than 3.5 mm	**14**
13b	Whorl diameter less than 3.5 mm	**15**
14a	Multiple ridges on dorsolateral edge of basal scales; Antarctica	*** N. gaussi ***
14b	Single ridge on dorsolateral edge of basal scales; Hawaiian Islands	*** N. bowersi ***
15a	Medial and buccal scales ridged; northwest Atlantic Ocean	*** N. regularis ***
15b	Medials and buccals not ridged; Indonesian region	*** N. parva ***
16a	Coenenchymal scales unridged (granular)(Fig. 2C, D)	*** N. valentine ***
16b	Coenenchymal scales ridged (Fig. 2E–G)	**17**
17a	Polyps 2.6–2.8 mm in length; South Pacific	*** N. virgosa ***
17b	Polyps 2.0–2.2 mm in length; Northwest Atlantic	*** N. bellissima ***
18a	Colony unbranched; single ridge on dorsolateral edge of basal scales	*** N. spectabilis ***
18b	Colony sparsely dichotomous; multiple ridges on dorsolateral edge of basal scales	*** N. abyssalis ***
19a	Four pairs of body wall scales per polyp (Fig. 1G)	*** N. laxa ***
19b	Three pairs of body wall scales per polyp	**20**
20a	Polychaete commensalism present, causing extreme modification of basal scales to form a tube	**21**
20b	Polychaete commensalism absent (no tubes)	**32**
21a	Branches of colony originate from a common base or from a basal bolus (Fig. 1K, L)	**22**
21b	Branching sparse, dichotomous (Fig. 1M)	**23**
21c	Branching equal, dichotomous (Fig. 1N)	**26**
21d	Branching pattern unknown; margin of basolateral scales tall and serrate	*** N. orientalis ***
22a	Body wall scales massive (Figs 1P, 2A); coenenchymal scales mosaic in arrangement; margin of basal scale spinose	*** N. horrida ***
22b	Body wall scales thin (normal); coenenchymal scales imbricate; margin of basal scale serrate	*** N. hypsocalyx ***
23a	Coenenchymal scales thick (mosaic) and unridged (Fig. 2C, D)	**24**
23b	Coenenchymal scales thin and ridged	**25**
24a	Body wall scales massive; numerous small adaxial buccal scales; western Pacific (Figs 1P, 2A)	*** N. clavata ***
24b	Body wall scales thin; 3 pairs of large adaxial buccals (Fig. 2J); eastern Pacific	*** N. ambigua ***
25a	Polyps 2.8–3.2 mm in length; distal margin of basal scales lobate and smooth (Fig. 1C)	*** N. aurantiacus ***
25b	Polyps 2.0–2.5 mm in length; distal margin of basal scales a serrate cowl (Fig. 2K)	*** N. leilae ***
26a	Coenenchymal scales ridged (Fig. 2E–G)	**27**
26b	Coenenchymal scales not ridged (Fig. 2C, D)	**29**
27a	Coenenchymal scales thin and imbricate in arrangement; polyps 2.5–3.1 mm in length	*** N. alata ***
27b	Coenenchymal scales thick (Fig. 2D) and mosaic in arrangement; polyps 1.8–2.0 mm in length	*** N. vermifera ***
28a	Whorl diameter more than 6 mm	**29**
28b	Whorl diameter less than 6 mm	**30**
29a	Polyp length 2.7–2.8 mm; few adaxial scales	*** N. obscura ***
29b	Polyp length 1.4–1.9 mm; numerous small adaxial scales (Fig. 2J)	*** N. dampieri ***
30a	Polyp length 2.7–3.1 mm; adaxial scales not ridged	*** N. mosaica ***
30b	Polyp length 2.0–2.4 mm; adaxial scales ridged	*** N. vulgaris ***
31a	Colonies unbranched	**32**
31b	Branching sparse, dichotomous	**33**
31c	Branching equal, dichotomous	**34**
31d	Branching lyrate, sometimes with subsequent dichotomous branching	*** N. compressa ***
32a	Polyp length 4.5–5.0 mm; distal margin of basal scales serrate	*** N. calamus ***
32b	Polyp length 3.2–3.7 mm; distal margin of basal scales lobate and smooth	*** N. versluysi ***
33a	Coenenchymal scales thick and mosaic in arrangement; polyp length approximately 3 mm	*** N. grandiflora ***
33b	Coenenchymal scales thin and imbricate in arrangement; polyp length 2.0–2.2 mm	*** N. speighti ***
34a	Body wall scales massive (Fig. 1P)	**35**
34b	Body wall scales thin (normal)	**36**
35a	Medial scales in open position; polyp length 3.0–3.3 mm	*** N. studeri ***
35b	Medial scales in closed position (fused); polyp length 1.8–2.0 mm	*** N. biannulata ***
36a	Coenenchymal scales unridged (granular)	**37**
36b	Coenenchymal scales ridged	**38**
37a	Coenenchymal scales thick and mosaic in arrangement; South West Indian Ocean	*** N. candidae ***
37b	Coenenchymal scales thin and imbricate in arrangement; Japan	*** N. japonensis ***
38a	Polyps per whorl fewer than 5	*** N. dichotoma ***
38b	Polyps per whorl 5–8	*** N. megalepis ***
38c	Polyps per whorl more than 9	*** N. gigas ***

## Supplementary Material

XML Treatment for Narella
